# The Mirror Theory: Parallels between Open Angle and Angle Closure Glaucoma

**DOI:** 10.3390/life14091154

**Published:** 2024-09-12

**Authors:** Vasile Potop, Christiana Diana Maria Dragosloveanu, Alina Mihaela Ciocâlteu, Miruna Gabriela Burcel, Maria Cristina Marinescu, Dana Margareta Cornelia Dăscălescu

**Affiliations:** 1 Ophthalmology Discipline, Faculty of Dentistry, Carol Davila University of Medicine and Pharmacy, 020021 Bucharest, Romania; 2 Clinical Hospital for Ophthalmological Emergencies, 010464 Bucharest, Romania; 3 Brasov County Emergency Clinical Hospital, 500326 Brasov, Romania; 4 Discipline Physiology III, Faculty of Medicine, Carol Davila University of Medicine and Pharmacy, 020021 Bucharest, Romania

**Keywords:** primary open angle glaucoma, primary angle closure glaucoma, trabecular meshwork, intraocular pressure

## Abstract

Glaucoma is a widespread ophthalmological disease, with a high impact and frequent visual morbidity. While the physiopathology of the two types of primary glaucoma (open angle and angle closure) has been studied, there seems to be little relationship between the two. In this study, we gather clinical and preclinical data to support the idea that the two primary glaucomas are “mirrored” in terms of morphological parameters and disease physiopathology. In short, primary angle closure glaucoma (PACG) is associated with hyperopia and low axial length, and primary open angle glaucoma (POAG) is associated with myopia and high axial length. Moreover, in PACG and in primary angle closure or primary angle closure suspect cases, while there is extensive iridotrabecular contact, the intraocular pressure (IOP) is still maintained in the lower half of the normal range throughout the evolution of the disease, which suggests a baseline trabecular hyperfiltration in PACG. In the opposite case, myopic eyes with open angles and a higher risk of developing POAG often have a baseline IOP in the upper half of the normal range, suggesting a baseline trabecular hypofiltration. As we explore clinical, genetic and animal model data regarding these opposing aspects, we hypothesize the existence of a mirroring relationship between PACG and POAG. Defining the relationship between the two potentially blinding diseases, with a high prevalence worldwide, may aid in understanding the mechanisms better and refining diagnosis and treatment. Thus, our theory has been named the Mirror Theory of Primary Glaucomas.

## 1. Introduction

Glaucoma is the most common cause of irreversible blindness [1]. It is estimated that, in 2020, 76 million people had glaucoma worldwide. By 2040, the number is projected to increase to 112 million [2]. The main classification of glaucoma is based on the iris-trabecular angle, which divides the primary glaucomas into primary open angle glaucoma (POAG) and primary angle closure glaucoma (PACG) [3]. The pooled global prevalence, obtained from a recent meta-analysis, is 3.05% for POAG and 0.50% for PACG [2].

POAG has a higher prevalence compared to PACG. The lifetime risk of blindness is 10% in POAG and 25% in PACG. Despite a better understanding of PACG etiopathogenesis, the risk of blindness is higher [4], and while PACG is less common worldwide, it is responsible for half of all bilateral blindness cases [5].

While no relationship has been discussed in the literature between the two forms of primary glaucoma, we suggest a possible opposite, symmetrical distribution of the two entities and we propose the Mirror Theory of Primary Glaucoma. In this article, we present a review of data from the literature regarding the trabecular outflow function of the two types of primary glaucoma, in connection with other ocular parameters, such as the refractive error.

## 2. Glaucoma Classification and Etiopathogeny

The two types of glaucoma, open angle and angle closure, may be differentiated by the morphology of the anterior chamber angle. Glaucoma may be classified further into primary and secondary (when the systemic or ocular pathology that caused the glaucoma may be identified) [6]. In this study, we will focus on primary angle closure and open angle glaucoma.

The iridocorneal angle is a structure at the periphery of the anterior chamber of the eye, between the iris and the cornea—the anterior wall of the angle pertains to the sclera, the posterior wall is the anterior face of the iris, and between the two we may find the ciliary body and the trabecular meshwork (a filtering structure connecting the anterior chamber and Schlemm’s canal—a circular channel, situated in the scleral sulcus, with a lymphatic-like structure) [7,8,9]. This ocular structure is important in glaucoma classification and pathophysiology because it houses the two aqueous humor (AH) outflow pathways:

- The conventional pathway: AH flows through the trabecular meshwork to the Schlemm’s canal, then the collector channels, and reaches the episcleral veins [9];
- The uveoscleral pathway (responsible for about 10% of the outflow): AH circulates to the ciliary body surface, the iris root, reaching the suprachoroidal space and then is absorbed into the orbital veins [9,10].

Both types of glaucoma are caused by a decreased trabecular outflow of AH, however the mechanisms differ significantly. In PACG, there is contact between the peripheral iris and the trabecular meshwork, which impairs the capacity of AH to reach the trabecular meshwork and filter through. In POAG, due to a remodeling of the extracellular matrix and cell aging and death in the trabecular meshwork, there is increased resistance in the trabecular meshwork; therefore, the outflow is lowered [8].

## 3. Trabecular Function and Refractive Status in Patients with Angle Closure

Eyes that are at risk of acute angle closure have a particular anatomy: the anterior chamber is shallow, the axial length (AL) is lower than average, the cornea has a smaller diameter and a steeper curvature, the crystalline lens is thick and positioned anteriorly, and, most importantly, gonioscopy reveals varying degrees of iridotrabecular contact (ITC) [11]. In a comparative study including Asian patients, it was reported that 26.6% had a low axial length (defined as under 22.0 mm), while only 13.3% of patients with open angles (confirmed by gonioscopy) had a low axial length [12]. Another study found markedly different biometric measurements in PACG and in normal controls: on average, AL is 20.74 mm in PACG, compared to 22.67 mm in the controls, and anterior chamber depth (ACD) is 2.44 mm in PACG, and 3.18 mm in the normal controls [13] (see Table 1).

In acute angle closure, the intraocular pressure (IOP) rises to extreme values, up to 50–70 mmHg [4] in patients with closed angles; outside of an acute closure, IOP is most often normal [4]. Our theory postulates that in such situations, the trabecular meshwork has an increased outflow rate to offset the areas of ITC in which outflow is severely impaired. More research is needed in order to confirm this notion, however few studies focus on IOP in angle closure, outside of acute attacks. A prevalence study on a Japanese population revealed that half of the patients with glaucomatous optic neuropathy and occludable angles (defined in the study as having PACG) had normal IOP [24]. In a study on a Korean population, 60.6% of patients with PACG had normal IOP at their first ophthalmological consult, these patients were also more likely to have a hyperopic refractive error [25]. Furthermore, the refractive error most commonly found in PAC patients, hyperopia, has been linked to lower IOP in a multivariate linear regression analysis [26].

The study of genes involved in PACG strengthens the connection with particular risk factors. For instance, a genetic hallmark of decreased axial length was found as well as ones related to hyperopia, to anterior chamber depth, to pupillary reflexes and to oxidative stress in the retinal ganglion cells [27]; the anterior chamber depth is also a highly heritable trait [28]. Another study involving iris specimens collected during a trabeculectomy surgical procedure has shown that, compared to POAG patients, irises from eyes suffering from PACG express a significantly higher amount of several genes: COL1A1 (collagen type I alpha 1 chain), VEGFB, VEGFC and VEGFR2 (vascular endothelial growth factors B and C and the VEGF receptor 2) [15]. These particular genes shed a light on the complex mechanisms of PACG; as the collagen fibers are complexly connected to blood vessels in the human iris, they may influence each other’s upregulation and protein production. Moreover, the increased expression of VEGF genes may be a response to the tendency of a PACG eye to experience ischemia, and the increased expression of COL1A1 is mirrored by the clinical observation that the iris is more rigid in PACG eyes—meaning it is less responsive to miosis and mydriasis stimuli [15]. In connection, in Genome-wide association studies (GWASs)—studies that compare the genotype of a disease to a normal control, identifying a statistically significant array of small differences, most often single-nucleotide polymorphisms, which correlate with a trait of interest which is not usually caused by a monogenic mutation [29]—the CHAT gene (encoding the enzyme choline O-acetyltransferase), a gene which modulates the pupillary response of miosis, has also been associated with PACG [16].

### Refractive Status in Angle Closure Glaucoma

PACG is associated with hyperopia: patients with low hyperopia (spherical equivalent 1.00–2.99 D) have a 2.82 higher risk of suffering from PACG compared to the emmetropic control population, while those with higher hyperopia (spherical equivalent ≥3.01 D) have a 7.74 times higher risk of PACG [30]. The anatomical basis of this connection is the narrow, occludable iridotrabecular angle; the prevalence of at least one quadrant of ITC is 7.43% in hyperopes, compared to 1.25% in a similar population of myopes [31]. This characteristic of hyperopic eyes correlates well with a lower axial length, a thick lens and a shallow anterior chamber [31,32].

One ocular structure involved in accommodation are the zonules—the contraction of ciliary muscles leads to the relaxation of anterior zonules, and thus a change in lens shape and an increase in the dioptric power of the eye in order to facilitate near work [33]. In hyperopes, accommodation is an intensely used mechanism that helps maintain clear vision and avoid asthenopia [34]. In this context, one argument in favor of our theory is the fact that a drop in IOP during accommodation was proven in several studies; in healthy subjects, alternating between accommodation and non-accommodation led to a statistically significant IOP decrease of 0.7 mmHg [35]. Similarly, in young emmetropic and myopic subjects, accommodation leads to a statistically significant IOP drop of 1.86 ± 1.1 mmHg [36]. This effect may be explained by the complex physiological synergy between accommodation and trabecular filtration. The contraction of the ciliary muscle, which accompanies accommodation, causes a morphological shift in the trabecular meshwork and may cause a dilation of the Schlemm’s canal. Moreover, as trabecular meshwork cells have contractile properties, their contraction further facilitates trabecular outflow [37]. As hyperopic patients engage intensively in accommodation, the AH outflow is maintained at higher levels and the IOP is kept lower. Moreover, a decrease in axial length was noted after glaucoma surgery (trabeculectomy) [38], which further strengthens the connection between a lower IOP and the morphological changes related to hyperopia.

An interesting connection between accommodation and glaucoma physiopathology is the observation that the contraction of the ciliary muscle, which causes the accommodative response, pulls the choroid and retina forward, applying tension on the optic nerve head [39]. A study on rhesus monkeys (whose accommodative mechanism and anatomical apparatus is sufficiently similar to humans to accurately translate these results) revealed that ciliary muscle contraction exerts pulling forces both on the lens zonules, and on the peripheral choroid and retina—more precisely, from the ora serrata extending to 4–7 mm posterior to it [40,41]. In humans, it has been reported that, during accommodation, the zonule insertion is moved forward by 1.05 ± 0.07 mm, while the connected tissues—the choroid and retina—are stretched and pulled by approximately 1.05 mm [40,41]. However, as presbyopia evolves, the zonules and lens lose elasticity, while the ciliary muscle maintains its contractile force—continuing to exert tension on the optic nerve head [39,42]. Therefore, a hyperopic patient, engaging extensively in accommodation and developing presbyopia relatively earlier in life, may present a higher degree of strain on the optic nerve head, which could compound the risk of glaucomatous damage. As experimental confirmation of this physiological feat, a recent study, published in 2022, quantified the changes exerted on the optic nerve head by accommodation. By obtaining fundus photo images of healthy subjects, in an accommodated and unaccommodated state, it was proven that in accommodation a statistically significant choroidal thinning and centrifugal stretching occur around the optic nerve head. Moreover, these findings were significant both in young patients and in presbyopes [43].

Nanophthalmos is a rare developmental pathology in which we find a morphology similar to hyperopia, but to a higher degree—with low axial length, high refractive values and a shallow anterior chamber all present [44]. While AL is a defining feature of this condition, there is no generally accepted cut-off value. Many studies define nanophthalmos as having an AL that is at least two standard deviations lower than the average population, and others use cut-off values between 21.00 mm and 18.00 mm [45]. There is considerable genetic overlap between nanophthalmos and both hyperopia and angle closure—with mutations being identified in the NNO1 (nanophthalmos 1), and MFRP (membrane-type frizzled-related protein) genes [16].

These patients have highly occludable angles, and an argument in support of our theory is the fact that a study involving nanophthalmic children found that 97% had a normal IOP, even though up to a quarter had occludable angles on gonioscopy [44]. Another study following 144 patients with nanophthalmos found an average IOP of 18.38 mmHg, despite a prevalence of 88.7% of angle closure documented on the gonioscopy (it is relevant to mention that 35.7% of patients had a previous diagnosis of PACG) [46]. Lastly, a short case series of 11 nanophthalmic eyes revealed an average IOP of 20.2 mmHg, despite all the patients having either appositional or synechial angle closure [47]. The last study mentioned offers us a clue towards our Mirror Theory; all the patients with a normal IOP (e.g., under 21 mmHg) had angle closure in approximately one quadrant, while those eyes with a high IOP had an extensive degree of angle closure—supporting the idea that, to a certain extent, the hyperfunctioning trabecular meshwork can maintain a normal IOP despite ITC [47].

## 4. Trabecular Function and Refractive Status in Patients with Open Angle

To mirror the clinical picture of angle closure glaucoma described above, our theory postulates open angle glaucoma is associated with an impaired trabecular function and myopia. POAG is caused by an increase in trabecular outflow resistance, due to the impaired metabolism of the extracellular matrix, which leads to its accumulation in the trabecular meshwork [48]. In terms of biometric measurements, it was reported that POAG patients have significantly lower lens vaults, a higher ACD and a higher anterior chamber volume, when compared with PACG patients [15]. Thus, in opposition to the postulated closed angle, and the hyperfunctioning filtration in PACG patients, in POAG patients we find a wide-open angle and a hypofunctioning trabecular meshwork.

To mirror the morphological and biometric data previously described for PACG, both axial length and anterior chamber depth are parameters which significantly differ from the normal population [19,21,23] (see Table 1). In a hospital-based cohort, patients with POAG had significantly higher ALs (23.16 mm) compared to the normal controls (22.69 mm). Similarly, the anterior chamber was significantly deeper (3.05 mm) when compared to the controls (2.86 mm) [19]. In an outpatient clinic-based study, the average AL of POAG patients was 24.4 mm; however, without significant differences from the controls [21]. Lastly, in a large, population-based study, the average AL was 23.7 mm, again comparable to that of the controls [23]. In a direct comparison of POAG and PACG, all these parameters were significantly different: average ACD was 3.47 mm in POAG and 2.81 mm in PACG, average lens thickness was 4.22 mm in POAG and 4.53 mm in PACG and lastly AL was on average 24.48 mm in POAG and 22.75 mm in PACG [20]. Lastly, in connection with our theory, which postulates that in POAG the trabecular meshwork is primarily hypofunctioning while in PACG it is hyperfunctional and only leads to IOP spikes when significant iridotrabecular contact occurs, anterior segment OCT reveals ITC in 87.60% of PACG eyes and in 0% of POAG eyes, in a cohort of uncontrolled hypertensive cases [20].

In terms of pharmacological interventions, this increased trabecular outflow resistance can be addressed using a novel therapeutic class: Netarsudil, a rho-kinase inhibitor, that was approved in 2019 in the European Union, and is indicated in open-angle glaucoma and ocular hypertension cases [49]. All other topical treatments lower IOP by either increasing uveoscleral outflow, or by decreasing aqueous humor production [50], while Rho kinase inhibitors increase the permeability of the Schlemm’s canal endothelial wall and induce relaxation of the smooth muscle fibers in the trabecular meshwork [51].

The connection between myopia and POAG has been widely studied. A recent meta-analysis revealed that a 1 Diopter increase in myopia brings a 20% increase in open-angle glaucoma risk, while in high myopes the risk increases steeply, with an odds ratio of 4.14 [52]. More precisely, the axial length is most connected to the increased risk of POAG—a one millimeter increase in AL increases the risk of POAG by 50% [53]. Another meta-analysis reveals that low myopia entails a 59% higher risk of open angle glaucoma, and moderate-high myopia carries a 2.92 times higher risk [54]. This increased risk is all the more relevant as myopia is projected to affect half of the population by the year 2040 [55].

It has been postulated that the optic nerve head is more vulnerable to glaucomatous damage due to the high axial length and thin sclera we find in myopia, while choroidal and retinal pigment epithelium atrophy may increase the damage caused by oxidative stress [52]. Peripapillary atrophy found in the beta zone has also been described both in glaucomatous neuropathy and in progressive myopia [56]. Outside of glaucoma, there have been reports that the IOP in myopia is higher on average [57,58]. A study has found that in high myopia (>−6.0 D) IOP is higher compared to medium myopia (−3.0 D to −6.0 D), the difference being statistically significant: 16.19 mmHg versus 15.51 mmHg in the high and moderate myopia groups, respectively [57]. In a pediatric study, the average IOP in the myopia group was 15.22 mmHg, while in the emmetropia group it was 13.35 mmHg, the difference being statistically significant. Moreover, there was a correlation between average increase in IOP and myopia degree, reaching an average of 19.21 mmHg in high myopia [58].

The role of accommodation is equally significant in myopia as it is in hyperopia; prolonged near work (all activities which require viewing an object at a close distance and therefore, engaging accommodation extensively [59]) has proven to be an important risk factor for progression [60]. In contrast with patients with stable refraction, in which accommodative demand results in an IOP decrease, a study reports that in progressing myopes accommodation is followed by a temporary IOP elevation [61].

Further, lowering IOP is being suggested as a potential therapy against myopic progression. Axial elongation may be due to scleral remodeling in response to IOP variation, and IOP lowering may impact fibroblast activity, matrix metalloproteinase activity and collagen degradation [62]. Moreover, myopic eyes have a lower choroidal thickness, and certain glaucoma medications have been proven to increase choroidal blood flow and thickness [62]. In an animal model of myopia, increasing choroidal blood flow with prazosin inhibited the axial lengthening and scleral hypoxia [63]. In another animal model of myopia, topical latanoprost, a glaucoma medication, significantly lowered the axial progression [64].

Several studies have reported pivotal connections between myopia and IOP [60,65,66,67,68]. While an older study found a significantly higher myopia progression rate in children with IOP over 16 mmHg when compared to children with IOP under 16 mmHg [67], a recent study has paradoxically found a negative association between axial elongation and IOP in children [68]. However, the authors attribute this peculiar relationship to the confounding effect of increased corneal compliance in high myopia, which will greatly influence IOP measurement and not accurately reflect the IOP [68]—having been shown previously that a myopic cornea has altered biomechanics compared to an emmetropic cornea [69]. In the same study, the AL (measured at one point in time, not the annual axial elongation) and IOP have an expected positive association, which decreases in significance as children age [68]. A study involving highly myopic adults, which did not evaluate IOP, has shown that not using intraocular pressure-lowering medications is associated with a faster axial length increase [65]. Lastly, a large study involving congenital glaucoma patients showed that the AL growth follows a steeper curve in children with IOP  >  21 mmHg compared to IOP ≤  21 mmHg, which plateaus around the age of three [66].

One of the best-known genes involved in POAG pathogenesis is the TIGR/myocilin gene, which is expressed in several ocular tissues, including the trabecular meshwork, where it decreases the AH outflow. Moreover, mutations in this gene might contribute to the heightened IOP increase as a response to glucocorticoid therapy [70]; although, it is known that a high axial length is a risk factor for steroid-response high IOP [71]. Research supports the idea that this genetic pathway contributes to the connection between POAG and myopia, as polymorphisms in the TIGR/myocilin gene have been associated with high myopia in population studies [72]. This was found not only in monogenic mutations, but also in a genome-wide association study, where a genetic correlation was found between POAG and both myopia and high myopia, showcasing a shared genetic basis [17].

## 5. Conclusions

The Mirror Theory of Primary Glaucoma suggests a common framework on which to understand the pathophysiology of primary glaucoma and to personalize treatment and follow-up for our patients. The central node of the theory is trabecular function, which is key in understanding the way PACG and POAG differ: in PACG, the baseline is hyperfiltration, which maintains normal IOP for a long period of time, and as iridotrabecular contact expands circumferentially, this compensation mechanism is overrun and IOP rises to very high values during an acute angle closure. This tableau is also developmentally associated with morphological factors such as low axial length, hyperopia and a shallow anterior chamber. Our theory suggests that an optimal therapeutic approach is keeping the eye in the initial state before extensive iridotrabecular contact.

Mirroring this situation, in POAG the baseline function of the trabecular meshwork is low: the impaired extracellular matrix metabolism leads to a high resistance and low outflow. This is associated, during the development of the eye, with an open angle, with no iridotrabecular contact, a higher axial length, a deep anterior chamber and myopia. Optimal therapy may include increasing trabecular outflow, which is a domain of POAG research that is currently being expanded with the addition of Rho kinase inhibitors; however, more research is needed.

## Figures and Tables

**Table 1:** Average values from the recent literature showcasing essential POAG and PACG parameters.

	Primary Open Angle Glaucoma (POAG)	Primary Angle Closure Glaucoma (PACG)
Prevalence (%)	3.05 [2]	0.50 [2]
Genetic risk factors	myocilin (MYOC)—involved in trabecular meshwork extracellular matrix; SIX Homeobox 6 (SIX6/606326)—associated with myopia; optineurin (OPTN)—involved in apoptosis of retinal ganglion cells; TANK-binding kinase 1 (TBK1)—involved in inflammation and autophagy [14]	COL1A1 and COL11A1—involved in collagen structure of trabecular meshwork [15,16]; VEGFB, VEGFC and VEGFR2 (vascular endothelial growth factors B and C and the VEGF receptor 2) [15]; NNO1—associated with nanophthalmos and PACG [16]; CHAT—involved in pupillary constriction [16]
Spherical equivalent (D)	−0.71 [17]	+1.38 [18]
Anterior chamber depth (ACD) (mm)	3.05 [19]; 3.47 [20]	2.44 [13]; 2.81 [20]
Corneal thickness (μm)	519.5 [19]; 513.5 [21]	545.6 [22]
Iridotrabecular contact (% of cases measured on anterior segment OCT)	87.60 [20]	0 [20]
Axial length (AL) (mm)	23.16 [19]; 23.7 [23]; 24.4 [21]; 24.48 [20]	20.74 [13]; 22.75 [20]

## Data Availability

No new data were created or analyzed in this study.
